# Supplementary material for: Contemporary Cardiovascular Risk Assessment for Type 2 Diabetes Including Heart Failure as an Outcome: The Fremantle Diabetes Study Phase II
Source: J Clin Med. 2020 May 11;9(5):1428. doi: 10.3390/jcm9051428 (PMC7290870; doi:10.3390/jcm9051428)
Supplement: Supplementary file 1 [file jcm-09-01428-s001.pdf]

Supplementary Materials

Table S1. Baseline characteristics of people with type 2 diabetes in Fremantle Diabetes Study Phase II by 5-year incident cardiovascular disease status (non-fatal stroke, non-fatal myocardial infarction and cardiovascular death; 3-point MACE). Data are percentages, mean ± SD, geometric mean (SD range) or median [inter-quartile range].

	No incident 3-point MACE	Incident 3-point MACE	*P*-value
Number (%)	1408 (90.8)	143 (9.2)	
Age (years)	65.3±11.4	69.2±13.3	0.001
Sex (% male)	51.6	54.5	0.54
Ethnic background (%):			<0.001
Anglo-Celt	53.6	51.0	
Southern European	12.5	14.0	
Other European	7.5	3.5	
Asian	4.6	2.8	
Aboriginal	5.7	12.7	
Mixed/other	16.1	11.9	
Not fluent in English (%)	10.6	11.2	0.78
Currently married/*de facto* relationship (%)	63.8	51.0	0.004
Educational attainment (%):			0.32
Some/completed primary or none	12.9	16.8	
Some/completed secondary	52.4	53.3	
Some/completed tertiary	34.7	29.9	
Any physical activity in past week (%)	90.1	75.8	<0.001
Smoking status (%)			0.044
Never	43.2	38.5	
Ex	46.9	44.8	
Current	9.9	16.8	
Alcohol consumption (standard drinks/day)	0.1 [0-1.2]	0.1 [0-1.5]	0.89
Age at diabetes diagnosis (years)	55.6±12.0	54.8±14.9	0.57
Diabetes duration (years)	8.0 [2.5-15.4]	15.0 [6.0-19.7]	<0.001
Diabetes treatment (%)			<0.001
Diet	25.1	14.0	
Oral hypoglycemic agents (OHAs)/non-insulin injectables	53.5	51.7	
Insulin only	5.1	10.5	
Insulin±OHAs/non-insulin injectables	16.3	23.8	
Fasting serum glucose (mmol/L)	7.5 (5.6-10.2)	7.7 (5.4-11.0)	0.43
HbA_{1c} (mmol/mol)	53 (41-68)	58 (43-78)	0.001

History of severe hypoglycaemia (%)	2.8	9.1	0.001
BMI (kg/m^2)	31.3±6.1	30.5±6.1	0.12
Central obesity (by waist circumference; %)	71.2	72.7	0.77
ABSI (m$^{11/6}$kg$^{-2/3}$)	0.081±0.005	0.083±0.005	<0.001
Systolic blood pressure (mmHg)	145±22	154±27	<0.001
Diastolic blood pressure (mmHg)	80±12	81±13	0.25
Pulse pressure (mmHg)	65±18	73±23	<0.001
Heart rate (bpm)	70±12	72±14	0.019
Orthostatic hypotension (%)	31.4	41.1	0.023
Taking antihypertensive medication (%)	72.9	83.8	0.005
Total serum cholesterol (mmol/L)	4.2 (3.3-5.4)	4.3 (3.2-5.8)	0.24
Serum HDL-cholesterol (mmol/L)	1.20 (0.92-1.55)	1.17 (0.89-1.55)	0.34
Non-HDL cholesterol (mmol/L)	2.9 (2.1-4.1)	3.1 (2.1-4.5)	0.17
Total:HDL-cholesterol ratio	3.5 (2.6-4.8)	3.7 (2.6-5.2)	0.055
Serum triglycerides (mmol/L)	1.5 (0.9-2.5)	1.6 (0.9-2.8)	0.14
Taking lipid-lowering medication (%)	68.3	71.1	0.51
Taking aspirin (%)	36.5	48.6	0.006
Atrial fibrillation/flutter (%)	4.2	8.6	0.032
Left ventricular hypertrophy (%)	1.4	7.1	<0.001
History of hospitalisation for/with rheumatic fever (%)	1.1	1.4	0.67
History of hospitalisation for/with heart failure (%)	5.4	17.5	<0.001
Cerebrovascular disease (%)	7.7	17.5	<0.001
Coronary heart disease (%)	25.7	56.6	<0.001
Peripheral arterial disease (%)	21.0	42.0	<0.001
Peripheral sensory neuropathy (%)	57.5	65.5	0.012
eGFR (CKD-EPI creatinine) category (%)			<0.001
≥90 ml/min/1.73m^2	39.3	27.5	
60-89 ml/min/1.73m^2	45.8	34.5	
45-59 ml/min/1.73m^2	8.5	14.8	
30-44 ml/min/1.73m^2	4.3	14.1	
<30 ml/min/1.73m^2	2.1	9.2	
Urinary albumin:creatinine ratio (mg/mmol)	3.0 (0.9-10.8)	8.3 (1.4-51.0)	<0.001
Any retinopathy (%)	35.7	51.1	0.001

Table S2. Baseline characteristics of people with type 2 diabetes in Fremantle Diabetes Study Phase II by 5-year incident cardiovascular disease (3-point MACE plus hospitalisation for/with heart failure (4-point MACE). Data are percentages, mean ± SD, geometric mean (SD range) or median [inter-quartile range].

	No incident 4-point MACE	Incident 4-point MACE	*P*-value
Number (%)	1306 (84.2)	245 (15.8)	
Age (years)	64.7±11.3	70.8±12.1	<0.001
Sex (% male)	50.8	58.0	0.043
Ethnic background (%):			<0.001
Anglo-Celt	53.1	54.3	
Southern European	12.4	13.9	
Other European	7.7	4.1	
Asian	4.9	2.0	
Aboriginal	5.7	12.7	
Mixed/other	16.2	13.1	
Not fluent in English (%)	10.4	11.8	0.50
Currently married/*de facto* relationship (%)	64.7	51.8	<0.001
Educational attainment (%):			0.002
Some/completed primary or none	12.1	19.6	
Some/completed secondary	52.2	53.6	
Some/completed tertiary	35.7	26.8	
Any physical activity in past week (%)	90.5	79.5	<0.001
Smoking status (%)			0.028
Never	44.0	36.3	
Ex	46.2	49.4	
Current	9.8	14.3	
Alcohol consumption (standard drinks/day)	0.1 [0-1.2]	0.1 [0-1.2]	0.21
Age at diabetes diagnosis (years)	55.4±11.8	56.3±14.5	0.33
Diabetes duration (years)	8.0 [2.0-15.0]	14.2 [6.0-19.8]	<0.001
Diabetes treatment (%)			<0.001
Diet	25.7	15.5	
Oral hypoglycemic agents (OHAs)/non-insulin injectables	54.4	47.8	
Insulin only	4.4	11.8	
Insulin±OHAs/non-insulin injectables	15.5	24.9	
Fasting serum glucose (mmol/L)	7.5 (5.6-10.1)	7.8 (5.5-11.0)	0.11
HbA_{1c} (mmol/mol)	53 (41-68)	57 (42-76)	<0.001
History of severe hypoglycaemia (%)	2.3	9.4	<0.001
BMI (kg/m^2)	31.2±6.0	31.4±6.8	0.73

Central obesity (by waist circumference; %)	71.0	73.4	0.49
ABSI ($m^{11/6}kg^{-2/3}$)	0.081±0.005	0.084±0.005	<0.001
Systolic blood pressure (mmHg)	145±21	151±28	0.001
Diastolic blood pressure (mmHg)	80±12	80±15	0.98
Pulse pressure (mmHg)	65±17	71±23	<0.001
Heart rate (bpm)	69±12	73±14	<0.001
Orthostatic hypotension (%)	31.3	37.6	0.07
Taking antihypertensive medication (%)	71.9	84.8	<0.001
Total serum cholesterol (mmol/L)	4.2 (3.3-5.4)	4.2 (3.2-5.6)	0.50
Serum HDL-cholesterol (mmol/L)	1.20 (0.93-1.55)	1.17 (0.88-1.55)	0.23
Non-HDL-cholesterol (mmol/L)	3.0 (2.1-4.1)	2.9 (2.0-4.2)	0.68
Total:HDL-cholesterol ratio	3.5 (2.6-4.8)	3.6 (2.6-4.9)	0.63
Serum triglycerides (mmol/L)	1.5 (0.9-2.5)	1.6 (0.9-2.6)	0.46
Taking lipid-lowering medication (%)	67.7	73.4	0.08
Taking aspirin (%)	35.5	48.8	<0.001
Atrial fibrillation/flutter (%)	3.3	11.7	<0.001
Left ventricular hypertrophy (%)	1.2	5.8	<0.001
History of hospitalisation for/with rheumatic fever (%)	0.9	2.0	0.17
History of hospitalisation for/with heart failure (%)	3.4	23.3	<0.001
Cerebrovascular disease (%)	7.0	17.1	<0.001
Coronary heart disease (%)	23.6	55.1	<0.001
Peripheral arterial disease (%)	20.2	37.1	<0.001
Peripheral sensory neuropathy (%)	56.0	72.2	<0.001
eGFR (CKD-EPI creatinine) category (%)			<0.001
≥90 ml/min/1.73m^2	41.4	21.3	
60-89 ml/min/1.73m^2	45.4	41.0	
45-59 ml/min/1.73m^2	8.4	12.7	
30-44 ml/min/1.73m^2	3.5	14.8	
<30 ml/min/1.73m^2	1.3	10.2	
Urinary albumin:creatinine ratio (mg/mmol)	2.8 (0.9-9.4)	8.0 (1.3-47.4)	<0.001
Any retinopathy (%)	34.5	51.3	<0.001

Supplementary Table S3. Examples of two clinical scenarios of 4-point MACE risk estimated using Model 4.

Clinical scenario	Linear risk function	The probability of an incident CVD2 event during the next five years
58.7 year old non-Aboriginal Australian man with diabetes duration 10.0 years, HbA_{1c} 51 mmol/mol, heart rate 58 bpm, uACR 0.54 mg/mmol, eGFR ≥45 ml/min/1.73m^2, PAD but no LVH, HF, or CVD	L = 0.0273 * (58.7 – 65.7) + 0.0006 * (58.7-65.7)^2 + 0.2924 * 1 + 0.5830 * 0 + 0.0162 * (10.0 – 10.2) + 0.5898 * (3.93-3.98) + 0.0173 * (58-70) + 0.1791 * (-0.61 – 1.22) + 0.6559 * 0 + 0.4006 * 1 + 1.0617 * 0 + 0.8602 * 0 + 0.7182 * 0 = -0.03678.	1-exp(-0.0478*exp(-0.03678)) = 0.045 (or 4.5%).
45.6 year old Aboriginal Australian woman with diabetes duration 13.0 years, HbA_{1c} 100 mmol/mol, heart rate 63 bpm, uACR 25.0 mg/mmol, eGFR ≥45 ml/min/1.73m^2, no PAD but LVH, HF, and CVD	L = 0.0273 * (45.6 – 65.7) + 0.0006 * (45.6-65.7)^2 + 0.2924 * 0 + 0.5830 * 1 + 0.0162 * (13.0 – 10.2) + 0.5898 * (4.61-3.98) + 0.0173 * (63-70) + 0.1791 * (3.22– 1.22) + 0.6559 * 0 + 0.4006 * 0 + 1.0617 * 1 + 0.8602 * 1 + 0.7182 * 1 = 3.5709.	1-exp(-0.0478*exp(3.5709)) = 0.817 (or 81.7%).

Table S4. Comparison of baseline characteristics of the Busselton Diabetes Study (BDS) external validation cohort and the Fremantle Diabetes Study Phase II (FDS2) cohort.

	BDS	FDS2	*P*-value
Number (%)	174	1551	
Age (years)	70.2±10.3	65·7±11.6	<0.001
Sex (% male)	51·7	51.9	>0.99
Aboriginal (%)	0	6.8	<0·001
Diabetes duration (years)	8.7 [5.0-14.1]	9.0 [3.0-15.9]	0.55
Heart rate (bpm)	69±12	70±13	0.52
LVH (%)	7.5	2.0	<0.001
Coronary heart disease and/or cerebrovascular disease (%)	32.2	33.1	0.87
Peripheral arterial disease (%)	5.7	22.9	<0.001
Ln(urinary albumin:creatinine ratio (mg/mmol))	3.0 (1.4-6.6)	3.3 (0.9-12.9)	0.39
eGFR <45 mL/min/1·73 m^2 (%)	8·0	8.0	>0.99
